# The Effect of Empagliflozin on Liver Fat in Type 2 Diabetes Mellitus Patients With Non-Alcoholic Fatty Liver Disease

**DOI:** 10.7759/cureus.16687

**Published:** 2021-07-28

**Authors:** Arbinda Pokharel, Sudhamshu KC, Pukar Thapa, Niyanta Karki, Rupesh Shrestha, Bikash Jaishi, Mukesh S Paudel

**Affiliations:** 1 Hepatology, National Academy of Medical Sciences, Kathmandu, NPL; 2 Gastroenterology, National Academy of Medical Sciences, Kathmandu, NPL

**Keywords:** nonalcoholic fatty liver disease (nafld), diabetes, fibroscan, empagliflozin, obesity

## Abstract

Background and objective

The prevalence of non-alcoholic fatty liver disease (NAFLD) is 60% in patients with type 2 diabetes mellitus (T2DM). NAFLD can lead to non-alcoholic steatohepatitis (NASH), both of which are the leading causes of cirrhosis. This study was undertaken to evaluate whether empagliflozin, a sodium-glucose cotransporter-2 (SGLT-2) inhibitor, reduces liver fat content in these patients after therapy.

Methods

After enrolling patients of T2DM with NAFLD, they were administered empagliflozin 10 mg once daily orally for six months without modifying existing oral hypoglycemic agents (OHA) if any. All demographic data were collected, and anthropometric measurements, as well as laboratory investigations, were performed, and controlled attenuation parameter (CAP) and liver stiffness (LS) were measured using FibroScan® (Echosens, Paris, France) at baseline, and six months of therapy. The adverse effects related to therapy were also taken into account.

Results

There was a significant decrease in mean CAP value from 282.07 ± 47.29 dB/m to 263.07 ± 49.93 dB/m and LS from 5.89 ± 4.23 kPa to 5.04 ± 1.49 kPa along with a significant decrease in serum alanine aminotransferase (ALT), aspartate aminotransferase (AST), and gamma-glutamyl transferase (GGT) among the patients. Compared to the baseline, there was a significant reduction in post-treatment weight, body mass index (BMI), and blood pressure (BP). The most commonly observed adverse effects of the therapy were urinary tract infection (UTI) (17.8%), nasopharyngitis (11.9%), and hypoglycemia (10.71%).

Conclusion

A reduction in hepatic fat content was seen in our prospective study cohort after six months of empagliflozin therapy. Empagliflozin also led to beneficial effects such as weight loss and reduction in transaminases and GGT. Given the absence of significant side effects of the therapy, empagliflozin could be used as an effective treatment modality for T2DM patients with NAFLD, which are two conditions commonly seen in combination.

## Introduction

Non-alcoholic fatty liver disease (NAFLD) is defined as hepatic steatosis diagnosed by histology or imaging with macrovesicular steatosis in >5% of hepatocytes [1]. Worldwide, the prevalence of NAFLD is 59.67% in patients with type-2 diabetes mellitus (T2DM). Studies from Nepal have reported the prevalence to be as high as 75% [2-4].

A novel parameter to assess steatosis is by a vibration-controlled transient elastography device, which measures controlled attenuation parameter (CAP). It measures the increased attenuation of ultrasound waves when traveling through steatotic hepatic tissue, compared to normal liver. It is non-invasive, quantitative, and non-ionizing [5,6]. It has been shown to efficiently detect steatosis at a rate of ≥10% [7].

Empagliflozin, a sodium-glucose cotransporter-2 (SGLT-2) inhibitor, improves glycemic control in patients with T2DM when used as monotherapy or in combination with other oral hypoglycemic agents (OHA), and weight loss is also a consistent feature associated with this medication [8,9]. In diabetic patients, most of the weight loss during treatment with empagliflozin occurs due to fat loss: abdominal visceral adipose tissue and subcutaneous adipose tissue [10-12]. Weight loss is the mainstay of the treatment of NAFLD or even non-alcoholic steatohepatitis (NASH). Loss of liver fat, as well as weight, can stop the progression of NAFLD to NASH. This study was carried out to examine the role of empagliflozin in lowering liver fat in Nepali T2DM patients with NAFLD.

## Materials and methods

This was a hospital-based prospective cohort study carried out in the Liver Unit, National Academy of Medical Sciences (NAMS), Bir Hospital, Kathmandu for a duration of six months (December 15, 2020, to June 15, 2021). Ethical approval was obtained from the Institutional Review Board of NAMS (approval number: 547-077/078). Patients aged more than 18 years with documented hepatic steatosis (ultrasound evidence of fatty liver) with uncontrolled T2DM defined by a glycated hemoglobin (HbA1c) >7.0% or recently diagnosed T2DM were included in the study. Informed written consent was obtained from the patients and/or their guardians.

Detailed history, physical examination, and systemic examination were performed. Patients were categorized based on their BMI as follows: normal - BMI from 18.5 to 24.99 kg/m^2^, overweight - BMI from >25 to 30 kg/m^2,^ and obese - BMI of >30 kg/m^2^. Baseline laboratory investigations included the following tests: complete blood count (CBC), renal function test (RFT), liver function test (LFT), and liver biochemistry that included total bilirubin (TB), direct bilirubin (DB), alanine aminotransferase (ALT), aspartate aminotransferase (AST), alkaline phosphatase (ALP), gamma-glutamyl transferase (GGT), prothrombin time (PT), and international normalized ratio (INR). Along with HbA1c, fasting blood sugar (FBS) and post-prandial blood sugar (PPBS), fasting lipid profile that included total cholesterol, triglycerides (TG), high-density lipoprotein (HDL) cholesterol, low-density lipoprotein (LDL) cholesterol, and urine analysis were also done.

CAP values and liver stiffness (LS) levels were obtained using FibroScan® (Echosens, Paris, France), which was performed by hepatologists with experience of at least five years in the field of hepatology. It was performed after overnight fasting with the patients in the supine position. At least 10 valid measurements were obtained for each participant. A success rate of ≥60% (the number of validated measurements divided by the total number of measurements) and a ratio of the interquartile range (IQR) of LS to the median (IQR/MLSM) of ≤30% was considered reliable and used for the final analysis. CAP was considered a valid value only when the LS for the same signal was reliable, using the same volume of liver parenchyma as in the LS.

CAP cutoff values with regard to liver steatosis (S) (which range from S1 indicating mild steatosis to S3 indicating severe steatosis) as given by the manufacturer are as follows: 230 dB/m for mild steatosis, 275 dB/m for moderate steatosis, and 300 dB/m for severe steatosis. The cutoff value for LS with the grade of fibrosis were taken as follows: <5.5 kPa for F0 (no fibrosis), 5.5-8.0 kPa for F1 (mild fibrosis), 8.0-10.0 kPa for F2 (moderate fibrosis), 11.0-16.0 kPa for F3 (severe fibrosis), and >16.0 kPa for F4 (cirrhosis) [13].

The patients were given empagliflozin 10 mg tablet once a day with breakfast. No changes were made to the existing OHA taken by the patients if any. Anthropometric measurements and laboratory parameters along with CAP and LS measurements were done after six months of therapy. Any adverse effects related to the therapy during the study period were also taken into account and managed as per the protocol. If necessary, the drug was withdrawn. Once the side effects were managed, the drug was reintroduced.

Data were collected using a structured proforma covering the relevant details. Continuous data were expressed as mean ± standard deviation (SD), median (IQR), and categorical variables were presented as numbers (%). In the case of continuous variables, the t-test/Wilcoxon rank-sum test was applied as appropriate to see the difference in average between the groups. The Chi-square test was used to see the association of categorical variables with outcomes. A p-value of <0.05 was considered statistically significant. Data were analyzed using SPSS Statistics version 20 (IBM, Armonk, NY).

## Results

Of the 84 patients included in the study, 68 (81%) were males and 16 (19%) were females. The majority of the patients belonged to the age group of 41-50 years (42.86%). The mean age of the patients was 47.23 ± 10 years (Table 1).

**Table 1:** Demographic profile of the patients

Characteristics	Category	N (%)
Sex	Male	68 (81)
	Female	16 (19)
Age group (years)	≤40	26 (30.95)
	41–50	36 (42.86)
	51–60	14 (16.67)
	>60	8 (9.52)

The baseline and post-therapy mean weight of the patients was 75.15 ± 8.43 kg and 72.37 ± 8.46 kg respectively. The mean reduction of weight was 2.27 kg (p=0.002). The pre-treatment BMI was 31.40 ± 3.31 kg/m^2^ and the post-treatment BMI was 30.29 ± 4.28 kg/m^2^, with a mean difference of 1.11 kg/m^2^, which was statistically significant; however, post-treatment BMI was still in the overweight category. The mean reduction of systolic blood pressure (SBP) and diastolic blood pressure (DBP), pre- and post-therapy, were 7.56 mmHg and 3.57 mmHg respectively, which was statistically significant (Table 2).

**Table 2:** Comparison of clinical characteristics BMI: body mass index; SBP: systolic blood pressure; DBP: diastolic blood pressure; SD: standard deviation

Parameters	Baseline (mean ± SD)	Post-treatment (mean ± SD)	Mean difference	P-value
Weight (kg)	75.15 ± 8.43	72.37 ± 8.46	2.78	0.002
BMI (kg/m^2^)	31.40 ± 3.31	30.29 ± 4.28	1.11	0.038
SBP (mmHg)	123.57 ± 13.68	116.01 ± 14.22	7.56	0.001
DBP (mmHg)	77.50 ± 8.49	73.93 ± 6.95	3.57	0.005

The mean HbA1c values, pre- and post-treatment, were 8.19 ± 0.93% and 7.50 ± 0.89% respectively, and the mean reduction was 0.69%, which was statistically significant. The mean FBS was 167.10 ± 48.21 mg/dL and 134.21 ± 49.77 mg/dL at baseline and post-treatment respectively. The mean difference was 32.88 mg/dL and the reduction of FBS was significant. The mean difference of PPBS was 64.83 mg/dL between pre- and post-therapy, which was also significant. Post-treatment AST and ALT decreased significantly compared to pre-treatment levels. There was also a significant reduction in GGT and TG post-therapy (Table 3).

**Table 3:** Comparison of biochemical profiles *Paired t-test; **Wilcoxon signed-rank test SD: standard deviation; HbA1c: glycated hemoglobin

Parameters	Baseline (mean ± SD)	Post-treatment (mean ± SD)	Mean difference	P-value
HbA1c (%)	8.19 ± 0.93	7.50 ± 0.89	0.69	0.000*
Fasting blood sugar (mg/dL)	167.10 ± 48.21	134.21 ± 49.77	32.88	0.000**
Post-prandial blood sugar (mg/dL)	308.40 ± 102.94	243.57 ± 86.93	64.83	0.000**
Alanine aminotransferase (IU/mL)	61.21 ± 30.66	50.48 ± 50.54	10.74	0.000**
Aspartate aminotransferase (IU/mL)	47.38 ± 20.22	42.15 ± 16.01	5.23	0.004**
Gamma-glutamyl transpeptidase (IU/mL)	136.93 ± 73.19	130.68 ± 70.11	6.25	0.000**
Triglyceride (mg/dL)	196.05 ± 93.31	172.37 ± 94.59	23.68	0.048**

The pre-treatment mean CAP value was 282.07 ± 47.29 dB/m and the mean post-treatment CAP was 263.07 ± 49.93 dB/m. The mean difference after treatment was 19 dB/m, which was statistically significant. LS also decreased significantly from pre-treatment (5.89 ± 4.23 kPa) to post-treatment (5.04 ± 1.49 kPa) with the mean difference of 0.85 kPa (Table 4).

**Table 4:** Comparison of ultrasound grading of controlled attenuation parameters and liver stiffness CAP: controlled attenuation parameter; LS: liver stiffness

Parameters	Baseline (mean ± SD)	Post-treatment (mean ± SD)	Mean difference	P-value
CAP (dB/m)	282.07 ± 47.29	263.07 ± 49.93	19	0.000
LS (kPa)	5.89 ± 4.23	5.04 ± 1.49	0.85	0.001

Regarding the side effects of the treatment, 17.8% of patients developed urinary tract infection (UTI), followed by nasopharyngitis (11.9%), and hypoglycemia (10.71%). Other complications experienced by the patients are presented in Table 5.

**Table 5:** Adverse effects of treatment

Complications	N (%)
Urinary tract infection	15 (17.8)
Genital-mycotic infection	4 (4.76)
Dyslipidemia	4 (4.76)
Nasopharyngitis	10 (11.9)
Upper respiratory tract infection	7 (8.3)
Arthralgia	4 (4.76)
Hypoglycemia	9 (10.71)
Headache	5 (5.9)
Acute kidney injury	1 (1.19)
Total adverse events	59 (70.2)
No adverse events	25 (29.8)
Total patients	84 (100)

## Discussion

As NAFLD is a major cause of liver disease throughout the world, we conducted this study to identify the effect of empagliflozin on liver fat in Nepalese patients. To the best of our knowledge, this is the first study from Nepal looking at steatosis reduction in patients with fatty liver and T2DM with the use of empagliflozin. The mean age of the study population was 47.23 ± 10 years. There was a male preponderance with a male-to-female ratio of almost 4:1. There were 68 (81%) males and 16 (19%) females respectively.

Our study demonstrated a significant reduction in CAP from 282.07 ± 47.29 dB/m to 263.07 ± 49.93 dB/m (p=0.000) after six months of therapy. Studies have shown that the CAP correlates well with the percentage and grading of hepatic steatosis [13,14]. So, a post-treatment decrease in CAP score can be taken as a reduction of fat in the liver. A similar finding was noted in a study by Shimizu et al. where there was a significant decrease in CAP from 314 ± 61 to 290 ± 73 dB/m (p=0.0424) [15]. Studies by Chehrehgosha et al. and Taheri et al. have also shown a reduction in CAP values [16,17].

In this study, there was a reduction of LS from a baseline value of 5.89 ± 4.23 kPa to a post-therapy level of 5.04 ± 1.49 kPa (p=0.001). Similar findings were seen in studies by Shimizu et al. and Taheri et al. [15,17]. However, the exact mechanism by which empagliflozin reduces liver fibrosis is unknown. A decrease in liver fat reduces chronic inflammation, which halts the rates of progression of fibrosis and increases the regression of fibrosis [18]. Although not a very specific and reliable marker of liver injury, serum aminotransferases are indirect markers of liver necroinflammation [19,20]. Our study showed a significant reduction in serum ALT and AST. A similar reduction was seen in studies by Kuchay et al., Sattar et al., and Takase et al. [12,21,22]. Since there was a reduction in fat as evidenced by a reduction in CAP, this may be a plausible explanation for the reduction in inflammation, hence the reduction in transaminase as well as LS values.

Our study demonstrated significant weight loss in patients, the mean difference being 2.78 (p=0.002). A study by Kuchay et al. [12] has also shown a significant weight loss (p=0.001), and another study has also elicited similar findings [22]. In our study, there was a significant decrease in both SBP and DBP, where the mean difference between SBP and DBP was 7.56 mmHg and 3.57 mmHg respectively. Our findings are supported by various other studies [23,24]. The exact mechanism by which empagliflozin lowers BP is unknown, but studies have suggested that it could be the effect of weight loss and reduction in arterial stiffness [25].

There was a significant decrease in HBA1c, FBS, and PPBS in the present study; the mean differences were 0.6, 32.88, and 64.83 respectively (p=0.000). A similar study by Chehrehgosha et al. also showed a significant drop in HbA1c, FBS, and PPBS (p<0.001) [16]. A study by Kuchay et al. has also reported similar results [12].

In the setting of T2DM, hepatic fatty acid metabolism is altered, commonly leading to the accumulation of TG within hepatocytes, and leads to NAFLD. Our study showed that TG was significantly decreased post-therapy, with a mean difference of 23.68. In a study conducted by Lai et al., the decrease in TG level (p=0.057) was statistically insignificant [24]. A similar finding was observed in another study [15]. In our study, the decrease in TG was significant because 5.95% of the study population had dyslipidemia and were on lipid-modifying agents (statin: 3.57% and fibrate: 1.19%) along with lifestyle modification as we had not modified any existing therapies being taken by the patients.

In our study, adverse events developed in 70.2% of patients, but there were no fatalities. Various studies have reported adverse events from 68% to 90% [26,27]. This difference in findings could be due to the difference in sample size and study population. The most common side effect was UTI (17.8%). None of the patients had fatal UTI. A similar finding was demonstrated in another study [27]. The risk of UTI is due to excessive glycosuria. Nasopharyngitis was noted in 11.9% of patients, which is in line with the findings of other studies [28,29]. Hypoglycemia developed in 10.7% of patients in our study. It was managed by standard protocol. The development of hypoglycemia in other studies has been variable. A study by Neeland et al. reported a value as low as 4.5% while Kholer et al. reported hypoglycemia in 15.7% of patients [23,29]. After recovery, empagliflozin was reintroduced with modified OHA. There were no clinically significant adverse consequences of hypoglycemia. The varied results could be because we had added empagliflozin without modifying the current OHA regimen of the patients.

Upper respiratory tract infection was seen in 8.3% of patients. Other adverse effects in our study were genitalia mycotic infection, dyslipidemia, and arthralgia, each with an incidence rate of 4.76%. Headache and acute kidney injury (AKI) developed in 5.9% and 1.19% respectively. The results were comparable to other studies [22,26,28].

The major strength of this study lies in the fact that we engaged in the measurement of CAP via FibroScan, which provides a non-invasive, simple, reproducible, and accurate method of quantification of hepatic steatosis. The study was conducted in a real-world scenario, where patients were receiving standard treatment for T2DM and other comorbidities. This study has some limitations. Firstly, CAP only quantifies the liver fat, not the inflammation. Also, this was not a randomized controlled trial and patients were already receiving the standard of care for T2DM and other comorbidities.

## Conclusions

The measurement of CAP by transient elastography is a feasible and effective method for the measurement of the fat content in the liver. We conclude that empagliflozin significantly reduces liver fat in patients with T2DM and NAFLD when used as monotherapy or in combination with standard medical therapy, as shown by the reduction of CAP values post-treatment in our cohort. Empagliflozin was also shown to have added benefits of reducing weight, as well as ALT, AST, and GGT levels. Given the absence of significant side effects of the therapy, empagliflozin could be used as an effective treatment modality for T2DM patients with NAFLD, which are two conditions commonly seen in combination.
